# Bacteriophages—a new hope or a huge problem in the food industry

**DOI:** 10.3934/microbiol.2019.4.324

**Published:** 2019-10-24

**Authors:** Marzena Połaska, Barbara Sokołowska

**Affiliations:** Institute of Agricultural and Food Biotechnology, Department of Microbiology, 36 Rakowiecka, 02-532 Warsaw, Poland

**Keywords:** bacteriophage, biosanitization, biopreservation, endolysins, biocontrol

## Abstract

Bacteriophages are viruses that are ubiquitous in nature and infect only bacterial cells. These organisms are characterized by high specificity, an important feature that enables their use in the food industry. Phages are applied in three sectors in the food industry: primary production, biosanitization, and biopreservation. In biosanitization, phages or the enzymes that they produce are mainly used to prevent the formation of biofilms on the surface of equipment used in the production facilities. In the case of biopreservation, phages are used to extend the shelf life of products by combating pathogenic bacteria that spoil the food. Although phages are beneficial in controlling the food quality, they also have negative effects. For instance, the natural ability of phages that are specific to lactic acid bacteria to destroy the starter cultures in dairy production incurs huge financial losses to the dairy industry. In this paper, we discuss how bacteriophages can be either an effective weapon in the fight against bacteria or a bane negatively affecting the quality of food products depending on the type of industry they are used.

## 1. Introduction

Bacteriophages are bacterial viruses acknowledged as the most diverse and abundant biological forms of life. The population of phages is estimated at around 10^31^ in the biosphere and significantly determines the number of bacteria in various ecosystems [1]. In the twenties of the 20^th^ century, the first attempts to use bacteriophages in medicine were limited by the lack of knowledge about the mechanisms behind their infection. Additional disadvantages were the low survival of phages in the acidic environment (e.g., the human stomach) and the presence of endotoxins in the applied phage cocktails. Endotoxins are highly immunogenic, and their presence in very large quantities may cause septic shock via cytokine signaling resulting in multiple-organ failure or even death [2]. Unfortunately, the interaction between the mammalian immune system and bacteriophages is not well understood. The immune response has an essential impact on the effectiveness of phage therapy in animals/humans. Phage immunogenicity refers to the generation of specific antibodies against bacteriophage antigens, which is a challenge in phage therapy, mainly due to its effects on the pharmacokinetics and the possible side effects of phages (e.g., anaphylactic shocks) [3],[4]. In addition, bacteriophages show immunomodulatory activity and hence can affect the functioning of immune cells [4].

The initial interest of scientists in the bactericidal properties of phages gradually decreased with the development of antibiotic therapy. However, the global problem of multidrug resistance of bacteria has recently renewed the interest in the use of phages as a potential tool against bacterial pathogens. In the context of food safety, bacteriophages can be an effective and inexpensive weapon against food-borne pathogenic bacteria, but at the same time, they are a serious threat to the quality of dairy products. Therefore, it is essential to ensure global food safety and preserve the quality of food products [5].

## 2. Phages as protection agents in the food chain

In the era of the development of organic foods and with rising awareness of healthy eating, non chemical measures for food protection are becoming increasingly popular. Phage cocktails meet all the criteria to be recognized as a green technology for combating food-borne pathogenic and spoilage bacteria. The application of phages in the food chain also has several advantages as follows:

- Bacteriophages are highly specific and usually can infect only one species or one type of bacteria. Thus, the natural commensal microbiota in the gastrointestinal tract of humans and animals is not destroyed.
- No adverse or toxic effect on eukaryotic cells has been observed with the use of bacteriophages. Bacteriophages are ubiquitous and present in many food products and different types of soils and water sources.
- Phages do not change the sensory properties of food products.
- Bacteriophages are highly resistant to the stress created during food processing [6],[7].

Bacteriophages are mainly used in three sectors in the food industry to ensure food safety: primary production, biopreservation, and biosanitization [7]. In primary production, phage therapy is used through phage added at the preharvest stage of production during the growth of plants or animals to eliminate the probability of plant or animal disease. Phages can also be applied at the postharvest stage during food processing and packing for controlling contamination by potential pathogens. In biosanitization, phages are applied to prevent and reduce biofilms on the surface of the equipments. In biopreservation, bacteriophages are directly added to food products to extend the expiry date of food [6].

### 2.1. The use of bacteriophages in plant crops

Phages intended for use in agriculture should be strictly lytic, specific, stable in the environment in which they are applied, and display minimum transduction [6],[7]. The bacteriophage-based product Agriphage™, produced by OmniLytics Inc. to treat bacterial spot disease on crops, was the first phage-based product formally approved for use in agriculture by the US regulatory agencies (US Environ. Prot. Agency) in 2005 (Table 2).

Biocontrol of bacteria in an open environment is difficult due to the direct exposure of bacteriophages to adverse environmental factors such as ultraviolet (UV) rays, dryness, temperature fluctuations, and remains of chemical agents used for plant protection [8]. However, there are many studies confirming the effectiveness of phage cocktails against several phytobacteria such as *Erwinia* spp. (cause bacterial soft rot and fire blight of apple and pear; specific phage Y2), *Xanthomonas* spp. (cause bacterial spot of tomato, peach, and citrus, walnut blight, leaf blight of onion, and citrus canker; phage specific F8, ΦXaacA1, CP2, ΦXac2005-1, ccΦ13, ΦX44), *Pseudomonas syringae* pv. *phaseolicola* (causes halo blight of bean; phage specific Ph1, Ph2), and *Ralstonia solanacearum* (causes bacterial wilt of tomato and tobacco; phage specific RSL1) [9]–[13]. Studies on phage treatment of *Xanthomonas* have been carried out for many years [10],[14],[15]. Among these, some important conclusions were put forth in the study of Civerolo and Kiela. First, the initial level of the applied bacteriophages must be high enough to achieve effective control of phytobacteria. Second, phages function more efficiently when applied prior to the bacterial infection or at the early stage of infection. For example, the authors treated one group of peach foliage by *Xanthomonas campestris* pv. *pruni* phage 1 hour before bacterial inoculation and another group 24 hours before bacterial inoculation. They observed the following results: in the phage-untreated group, there were 58% of infected leaves; in the group treated by phage 1 hour before bacterial inoculation, there were 22% of infected leaves; and in the group treated by phage 24 hours before bacterial inoculation, there were 29% of infected leaves [10]. Scientists tested for example the sensitivity of *X. campestris* pv*. pruni* to phage F8 infection on the surface of nectarine fruitlets and the ability of F8 phage to survive in controlled climatic conditions of a chamber compared to uncontrolled conditions in orchards. After the treatment of fruits with the phage suspension, it was observed that the disease did not develop in 92% of the tested fruits. Researchers also noticed that the decrease in phage population in orchards was 10^4^ times greater than the decrease in the climatic chamber. The potential reason for such a large decrease in phage population in the natural environment was high temperature, dehydration, and UV radiation [14]. Subsequent studies also investigated the effectiveness of phage cocktails on *Xanthomonas*, with significant attention paid to the impact of environmental conditions on the effectiveness of phage therapy. Phage cocktails were found to be more effective in the reduction of the tomato bacterial spot caused by *Xanthomonas* sp. after suspension of phages in 0.75% powdered skim milk and a mixture of 0.5% pregelatinized corn flour containing 0.5% sucrose. Both formulas enhanced phage persistence by alleviating the effect of UV radiation and allowing rainfastness. Researchers also reported that the activity of phages is influenced by the time of their application. Early morning or late evening is usually considered as the best time of phage application due to the reduction of UV rays as these rays negatively the efficiency of phages [15]. Currently, scientists are in the search for an effective factor that can, for example, reduce the harmful effects of UV radiation on the bacteriophages used in agriculture. The mixtures of Y2 bacteriophage acting against *Erwinia amylovora* and one of the tested protective agents (natural extracts from red pepper, carrot, beetroot, casein, soy peptone in solution, Tween 80, or purified substances such as astaxanthin and aromatic amino acids) were exposed to UV rays (λ = 254 nm) for 30, 60, 120, or 300 s. All the tested compounds were found to significantly increase the half-life of the Y2 phages exposed to UV irradiation and had no negative impact on phage viability or caused infectivity. Although the results of the *in vitro* tests on the use of the above-described factors for protecting phages in the natural environment were promising, it is necessary to check their effect *in vivo* during prolonged exposure to natural UV radiation [13]. One of the latest published studies highlighted the effectiveness of bacteriophages against *P. syringae* in their *in vivo* studies. In these studies researches used aerosol of single-phage suspensions and a phage mixture containing 3% corn flour and 5% sucrose. Spraying the bean leaves before the inoculation with *P. syringae* was found to reduce the disease severity by 58.57–61.14% in the case of phages used individually and by 70.8% in the case of a mixture of two phages [11].

The stability of the bacteriophage cocktail with potential applications in the propagation of plant crops depends, for example, on the resistance of phages to adverse environmental factors. The choice of bacteriophage suitable for the growing conditions seems to be a key. A bacteriophage RSL1 against *R. solanaceum* showing resistance to high temperature (37–50 °C) has been found. Control tomato plants infected with *R. solanacearum* not exposed to mentioned above bacteriophage showed the first signs of wilting after 4 days. By contrast, tomato plants treated with RSL1 phages specific for *R. solanacearum* did not show any wilting symptom during the experiment [12].

Another important factor affecting the effectiveness of the bacteriophages used in agriculture is the species of percentage of susceptible strains relative to bacteriophage resistant strains in the environment [15],[16].

In case of fresh cut fruits, environmental conditions also affect on phage effectiveness. In the described studies the pH of fruits was demonstrated to have an impact on the stability and activity of phages. [17] The application of bacteriophages specific to *Salmonella* spp. was effective in the case of cut melons, and the bacterial population was reduced by 2.5 log at 20 °C and 3.5 log at 10 °C. By contrast, the same bacteriophage cocktail did not produce any reduction in the bacterial population in the case of apples. The researchers concluded that these results point to the inhibition of the tested phages by the low pH of apples [17].

### 2.2. The use of bacteriophages in livestock production

Phage therapy in livestock production might refer to both the prevention of infections by pathogens through the application of bacteriophage in the form of probiotic or the disinfection of the animals' skin prior to slaughter and the treatment of a disease already existing in animals using bacteriophage cocktails [6].

The bacteria and phage populations are highly dynamic over time and rapidly (co)evolving thus, the correlation between the rate of resistance development *in vivo* and *in vitro* is an area that requires further study. Bacteriophages can evolve to avoid recognition by CRISPR (clustered, regularly interspaced short palindromic regions) resistance. CRISPR/cas system is considered to be the one of the earliest inheritable defense system that has evolved in micorganisms [18]. The exposure of a certain bacterial strain to a single bacteriophage is suggested to aid in the emergence of phage-resistant strains of the bacteria. On the other hand, recent evidence suggests that phage cocktails help control or delay the evolution of phage-resistant strains [19].

#### 2.2.1 Animals phage therapy

In the literature, there are studies on the effectiveness of phage therapy tested mainly in four models of animals: poultry, cattle, sheep, and swine [20]–[24].

Poultry is the most frequently used model in research on phage therapy. The high efficiency of a cocktail composed of two bacteriophages (CP8 and CP3) that are lytic for *Campylobacter jejuni* was demonstrated. The experiment was carried out on 25-day old broiler chickens previously colonized with the isolates from *C. jejuni* broilers. Phage treatment caused a reduction in the counts of *C. jejuni* at the level of 0.5–5.0 log CFU/g of cecal contents compared to the untreated control. The efficiency of phage therapy was found to be dependent on the type and the dose of the bacteriophage applied [20]. According to Wagenaar et al. (2005), the preventive use of phage mixtures (phage strains 69 and 71) applied by oral gavage resulted in delayed colonization of the gastrointestinal tract of chickens by *C. jejuni*, and the colonization process stabilized within a week at levels comparable to those observed in the therapeutic group after an initial decline of 2.0 log. By contrast, the phage therapy initially reduced the counts of *C. jejuni* by 3.0 log after several days, while the count of *C. jejuni* was only 1 log less than that recorded in the untreated control group of chickens [21].

Described studies evaluated the effectiveness of sprayed phage in cattle hides in preventing the infections caused by *Escherichia coli* O157:H7. The experiment was carried outbefore processing in beef processing plants. The level of contamination by *E .coli* in the phage-untreated controls was 57.6%. In the case of phage-sprayed cattle hides, the level of *E. coli* contamination was slightly lower amounting to 51.8%. These results showed that the administration of bacteriophage in the form of spray before processing is ineffective as a significant reduction of *E. coli* O157:H7 was noted on cattle hides [22].

The ability of phage cocktail to reduce the population of *Salmonella enterica* serovar Typhimurium in pigs during transport and holding prior to slaughter was investigated. A bacteriophage cocktail was applied via oral gavage. The tested mixture was found to be effective in combating the infection of *Salmonella* in the intestines of pigs. The contamination of the cecum by *S. enterica* serovar Typhimurium was found to be 95% lower while the contamination of the ileum was lower by 90%, compared to the control group which received mock treatment [23].

Sheep farmers often have to deal with the problem of *E. coli* infection. Bach et al. (2009) described the potential use of bacteriophages in protecting sheeps against *E. coli* infection. On day 0, the experimental sheeps were contaminated four *E. coli* strains. The bacteriophage cocktail composed of three bacteriophages (10^10^ PFU each of P5, P8 and P11 ) was applied orally on days −2, −1, 0, 6, and 7 of the trial. The fecal samples were collected, and a significant reduction of *E. coli* O157:H7 was observed. The researchers also pointed out that the effectiveness of the phages should be improved by protecting them from the conditions prevailing in the digestive tract of animals such as the effects of low pH and digestive enzymes as these factors adversely affect the phage survival [24].

An interesting phage solution was tested for application in aquaculture. Researchers investigated the effect on bacteriophage-based edible antimicrobial coatings on fish feed. These coatings, which were made from whey protein isolate (WPI), improved the stability of bacteriophages on fish feed pellets and their survival during the storage of feed. The tested coatings decreased the release of phages in salt water to >1 log PFU/pellet compared to the control coatings without a biopolymer used on feed. Phage specific for *E. coli* and *Vibrio* spp. were incorporated with WPI coatings caused higher bacterial reduction (3.0–5.0 logs of target bacteria) in the simulated intestinal fluid [25].

#### 2.2.2 Encapsulation as a phage-protective method

In the investigated studies the effectiveness of bacteriophages e11/2 and e4/1c *in vivo* and *ex vivo* for use in the control of *E. coli* O157:H7 in cattle. The examined phages were found to be effective in the *ex vivo* rumen model. When the phage cocktail was applied orally, there was no significant difference noted between the treated and untreated cattle [26]. Oral application of bacteriophages is a huge challenge to scientists due to their high sensitivity to the low pH in the stomach and the action of digestive enzymes. The authors overcame this problem through the bacteriophage encapsulation technique. Encapsulation of microorganisms in a suitable coating (hydrocolloids) allowed the release of the phages at a controlled rate under the influence of appropriate factors [27]. The encapsulation method in which the phages were immobilized in skim milk powder and enclosed in capsules consisting of palmitic and stearic acid was patented. Capsules with phages were more resistant to pH 2.15 than the nonencapsulated bacteriophages [28]. Stanford et al. (2010) also arrived at the same conclusion in their study. Four encapsulated bacteriophages were used to control the infection of *E. coli* O157:H7 in cattle. A 13.6% rate of recovery of active bacteriophages was obtained in the group treated by encapsulated bacteriophages exposed to pH 3.0 for 20 min. In the case of nonencapsulated phages, a complete loss of activity was observed under similar acidic conditions [29]. Some researches pointed out the need of encapsulating phages that are intended to be administered orally to pigs. In these studies, all pigs were contaminated orally with *S. enterica* serovar Typhimurium [30]. Researchers compared the results of three groups of pigs—control untreated phage group, group treated by encapsulated phage cocktail administrated orally with feed, and the group that received the same phage cocktail by gavage. In the group treated with encapsulated phages, the content of *Salmonella* in the ileum was 2.0 log CFU/mL contents and in the cecum was 2.7 log CFU/mL. In the case of the control pigs, the level of *S.* Typhimurium in the ileum was 3.0 log CFU/mL contents and in the cecum was 3.7 log CFU/mL. Very high concentrations of anti-Salmonella phages were detected in ileal and cecal contents from feed and gavage pigs (feed ileal: 1.4 × 10^6^; feed cecal 8.5 × 10^6^; gavage ileal 2.0 × 10^4^; gavage cecal: 2.2 × 10^3^). The concentration of bacteriophages in ileal and cecal were 2.0–3.0 log lower for phages administrated by gavage [30]. Bacteriophages are most often administered with both feed and water, enclosed in alginate beads and additionally coated with various polymers. The strength of the alginate hydrogel structure is increased by the use of chitosan, whey protein, or xanthan gum. The encapsulation of bacteriophages in alginate capsules was first described in 2008 year [31]. The researchers enclosed *Salmonella*-specific bacteriophage FelixO1 in alginate capsules surrounded by chitosan. As a result, only partial protection of bacteriophages against gastric acid in the simulated gastric juice was noted, whereas the time of bacteriophage release in the simulated intestinal fluid was relatively slow and amounted to almost 5 hours. The addition of calcium carbonate to the alginate capsules improved the resistance of the phages to gastric acid but did not improve the release of phages in the gut [31].

### 2.3 Commercially available phage-based products against food-borne pathogens

Food is the primary route of transmission for more than 200 known diseases. The leading bacterial food-borne pathogens of concern are *Salmonella*, *Campylobacter*, Shiga toxin-producing *E. coli*, and *Listeria monocytogenes* [32]. Each of them can be associated with serious gastrointestinal infections. Food-borne diseases remain a major cause of hospitalization and death worldwide, despite many advances in modern technologies including food sanitation techniques and pathogen surveillance. As estimated by the World Health Organization (WHO), 600 million—almost one in 10 people in the world—fall ill after eating contaminated food and 420 000 die every year [33]. Several approaches are used to improve the safety of our foods, but food-borne outbreaks occur relatively frequently. A new multihurdle approach identified to prevent the food-borne bacterial pathogens from reaching the consumers is the use of lytic bacteriophages for targeting specific food-borne bacteria in foods, without deleteriously impacting their normal—and often beneficial—microflora. This approach is termed ‘bacteriophage or phage biocontrol’ [34]. Table 1 summarizes the list of studies on bacteriophage biocontrol of the more important food-borne pathogens.

Since the regulatory acceptance of the first phage-based product, ListShield™ (approved in 2006 as ‘generally recognized as safe’), for use in the control of *L. monocytogenes* in meat and poultry products, the attempts to develop new phage-based technologies for pathogen control in postharvest foods have increased [3],[35]. The main organizations approving bacteriophage cocktails for use in agri-food sector are the European Food Safety Authority (EFSA) and the US Food and Drug Administration (FDA).The production of commercially available bacteriophage cocktails should be carried out according to the good manufacturing practices. Bacteriophages used in phage-based products should be strictly lytic (use of lysogenic phages for phage therapy is undesirable due to horizontal gene transfer) and effective against the highest possible number of strains belonging to the target bacterium [8]. It is recommended to use cocktails consisting of a mixture of bacteriophages to achieve improved efficiency and avoid the formation of strains resistant to bacteriophages [3],[36]. Changes in the composition of phage cocktails should be made systematically; however, this may be associated with additional difficulties in the production of these cocktails. For example, according to the EU regulations (1107/2009 EC), changes in the composition of plant protection products based on bacteriophages may require a new registration [8].

*Campylobacter* infections are among the most frequently encountered food-borne bacterial infections around the world. Handling and consumption of raw or undercooked poultry products have been identified to be the main route of transmission of these infections. Studies have analyzed the use of phages to target the *Campylobacter* bacteria growing on the surface of chicken carcasses, raw chicken meat, and raw and cooked beef [37]–[39].

The Shiga toxin-producing *E. coli* serotype O157:H7 can invade the human gastrointestinal tract and trigger disease, with symptoms including abdominal cramping and hemorrhagic diarrhea. Recent work has demonstrated that *E. coli*-specific phage preparation was effective in inhibiting this serotype [41]–[46].

*Listeria monocytogenes* is a major food-borne pathogen of public health concern associated with a high mortality rate in individuals at risk such as pregnant women, neonates, immunocompromised individuals, and the elderly [61]. It is therefore critically important to ensure the safety of the food chain, especially in the case of ready-to-eat (RTE) foods. The application of bacteriophages to assorted foods has been shown to be effective at reducing contamination with *L. monocytogenes* (Table 1).

Food-borne *Salmonella* infections are a major public health concern worldwide. All *Salmonella* phages reported so far have been able to decrease the number of viable cells present in raw meats, processed and RTE foods, and fresh products but not on apple slices. This indicates that the acidic pH of the apples may have inactivated the phages [17].

The concept of hurdle technology has been applied in the food industry following the observations that the rate of microorganism survival decreases greatly when the organisms are confronted with multiple antimicrobial factors or hurdles [62]. Several studies demonstrated the synergistic effect of using a bacteriophage with another food-grade antimicrobial such as nisin [57] and *trans*-cinnamaldehyde oil [46]. The antibacterial effect was also reported to be improved when phages were combined with a protective culture or modified atmosphere packaging [45],[49].

The *Listeria*-specific phage was effective against *L. monocytogenes* when used alone, and additionally, enhanced the effectiveness of other antimicrobials such as sodium diacetate and potassium lactate when used together [51],[52].

In recent years, an increasing number of phage products have been commercially used for pathogen control, as shown in Table 2.

**Table 1.** Bacteriophage biocontrol of food-borne pathogens. Adapted and modified from Moye et al. (2018) [34] and Kazi and Annapure (2016) [6].

Target pathogen	Kind of food	Phage	Results of the study	Reference
*Campylobacter*	Chicken skin	NCTC 12674	2-log drop seen in frozen–thawed samples	[37]
			1.0-log drop seen in fresh samples	
	Chicken skin	NCTC 12673	1.0-log reduction seen in treated group compared to untreated group	[38]
	Raw and cooked beef	Cj6	*Campylobacter* levels significantly decreased	[39]
	Raw chicken meat	NCTC 12684, or CP81	No reduction in bacterial load seen at 4 °C	[40]
*Escherichia coli* O157:H7	Meat (beef surface)	Cocktail of three phage: e11/2, pp01, e4/1c	Eradication of 10^3^ CFU/g of *E. coli* in seven of nine samples	[41]
	Meat (ground beef)	Cocktail EcoShield™ (formerly ECP-100)	*E. coli* levels decreased by 1.0 log	[42]
	Vegetables (tomatoes, broccoli, spinach)		*E. coli* levels decreased by 1.0–3.0 log	
	Lettuce and cantaloupe		Significant reduction (1.9 and 2.5 log, respectively) after 2 days of spraying	[43]
	Beef and lettuce surface		Levels of *E. coli* reduced by >94% and 87% on the surface of beef and lettuce, respectively	[44]
	Leafy greens		Levels decreased by >2 log under both ambient and modified atmosphere packaging storage	[45]
	Vegetables (lettuce, spinach)	Cocktail BEC8	At various temperatures, the level of *E. coli* reduced by 2.0–4.0 logs. The essential oil (*trans*-cinnamaldehyde) increased this effect	[46]
	Spinach blade	Bacteriophages specific for *E.coli* O157 collected and isolated from feedlot cattle feces	4.5-log reduction of *E. coli* after 2 hours of phage addition	[47]
*Listeria monocytogenes*	Fresh-cut fruit	Mixtures of LM-103 (14 phages) and LMP-102 (6 phages)	Reduction of 2.0–4.6 log in melons and only 0.4 log in apples. Nisin increased this effect to 5.7 log in melons and 2.3 log in apples	[17]
	Surface-ripened red-smear soft cheese	PhageGuard Listex™ (formerly Listex™ P100)	Significant reduction (at least 3.5 log) or a complete eradication of Listeria viable counts	[48]
	Cooked ham		1-log reduction after 14–28 days of storage. The protective culture *Lactobacillus sakei* TH1increased this effect by 2.0 log	[49]
	Raw catfish fillets surface		Levels of *Listeria* decreased by 1.4–2.0 log at 4 °C, 1.7–2.1 log at 10 °C, and 1.6–2.3 log at 22 °C. Regrowth was not observed at 2 and 10 °C	[50]
	Quesofresco cheese		Counts of *Listeria* decreased by 3.0 log in quesofresco cheese; however, subsequent growth was observed. Regrowth was prevented when potassium lactate (PL) + sodium diacetate (SD) was included with the phage	[51]
	Roast beef and turkey		Single phage was more effective at decreasing *Listeria* levels than PL or SD; subsequent bacterial growth was observed. When PL or PL + SD was used combined with phage,regrowth was prevented or diminished	[52]
	RTE sliced pork ham		Reduction *Listeria* counts below the limit of detection and performed better than nisin, sodium lactate, or combination of these antibacterial measures	[53]
	White mould (Camembert-type) cheese Washed-rind cheese with a red-smear surface	A511	On Camembert-type cheese, viable counts dropped by 2.5 log at the end of the 21-day ripening period; on red-smear cheese ripened for 22 days, and *Listeria* counts reduced by more than 3 log	[54]
	Vacuum-packed RTE chicken	FWLLm1	Reduction by 2.5 log at 30 °C and then regrowth. At 5 °C, regrowth was prevented over 21 days	[55]
*Salmonella*	Cheddar cheese	SJ2	*Salmonella* levels were reduced by 1.0–2.0 log in raw and pasteurized cheese created using milk that was treated with phage. No survival during 89 days in pasteurized cheese	[56]
	Fresh-cut fruit	Four phage cocktail SCPLX-1	Significant reduction on fresh-cut melons (2.5–3.5 log) but not on apples	[57]
	Chicken frankfurters	Felix O1	Reduction of *Salmonella* by 1.8–2.1 log	[58]
	Raw and cooked beef	P7	Reduction of 3.0–4.0 log at 5 °C and 6.0 log at 24 °C	[59]
	Turkey deli meats	FO1-E2	5.0-log reduction at 15 °C and 3.0-log reduction at 8 °C	[59]
	Chocolate milk			
	Hot dogs		3.0-log reduction at 8 °C and 15 °C	
	Seafood			
	Pig skin	UAB_Phi 20, UAB_Phi 78, UAB_Phi 87	Significant reduction (>4 and 2 log/cm^2^ for *S.* Typhimurium and *S.* Enteritidis, respectively) at 33 °C for 6 hours	[60]
	Chicken breasts		Significant decreases by 2.2 and 0.9 log CFU/g at 4 °C for 7 days	
	Fresh eggs		Minor reduction by 0.9 log at 25 °C for 2 hours	
	Packaged lettuce		Significant reduction by 3.9 and 2.2 log CFU/g for *S.* Typhimurium and *S.* Enteritidis, respectively	

**Table 2.** Examples of commercially available bacteriophage products. Adapted and modified from Moye et al. (2018) [34] and de Melo et al. (2018) [36].

Manufacturer	Products	Target pathogen	References
OmniLytics Inc. / USA www.omnilytics.com	Agriphage™	*Xanthomonas campestris* pv. *vesicatoria, Pseudomonas syringae* pv. *tomato* Biological control for bacterial spot and bacterial speck on tomatoes and peppers	Registered in USA (EPA Reg. No. 67986-1)
	Agriphage CMM™	*Clavibacter michiganensis* subsp. m*ichiganensis* Biological control for bacterial canker on tomatoes	Registered in USA (EPA Reg. No. 67986-6)
	Agriphage-Fire Blight	*Erwinia amylovora* Biological control for fire blight on apples and pears	Registered in USA (EPA Reg. No. 67986-8)
	Agriphage-Citrus canker™	*Xanthomonas citri subsp. citri* Biological control for citrus canker on orange, grapefruit, pummelo, lemon, lime, tangerine, tangelo, or kumquat	Registered in USA (EPA Reg. No. 67986-9)
Intralytix Inc. / USA www.intralytix.com	ListShield™	*Listeria monocytogenes* contamination in foods and food processing facilities	USA (FDA 21 CFR § 172.785, FSIS Directive 7120.1, GRAS GRN No. 528), Health Canada (iLONO), National Food Service of Israel (Ref: 70275202)
	EcoShield™	*E. coli* O157:H7 contamination in foods and food processing facilities	USA (FDA FCN No. 1018, FSIS Directive 7120.1, Health Canada (iLONO), National Food Service of Israel (Ref: 70275202)
	SalmoFresh™	*Salmonella* spp. on red meat and poultry	FSIS Directive 7120.1, GRAS GRN No. 435), Health Canada (iLONO), National Food Service of Israel (Ref: 70275202)
	ShigaShield™	*Shigella* spp. including *S. flexneri*, *S. sonnei* and *S. dysenteriae* contamination in foods and food processing facilities; is specifically designed for treating RTE meat and poultry, fish (including smoked fish), shellfish, fresh and processed fruits and vegetables, and dairy products including cheese	GRAS GRN No. 000672
	Ecolicide™	*Escherichia coli* O157:H7 contamination in pet food	-
	SalmoLyse™	*Salmonella* contamination in pet food	-
	ListPhage™	*Listeria monocytogenes* contamination in pet food	-
	Ecolicide PX™	*Escherichia coli* O157:H7 contamination on hides of live animals	-
	PLSV-1™	Animal health care products effective against *Salmonella* in poultry	-
	INT-401™	Animal health care products effective against *Clostridium perfringens* in poultry	-
Elanco Food Solutions/USA www.elanco.us	Finalyse™	*Escherichia coli* O157:H7 - the first pre-harvest hide wash for live cattle	-
Micreos Food Safety/Nederlands www.micreos.com www.phageguard.com	PhageGuard Listex™ (formerly Listex™P10)	*Listeria monocytogenes* surface intervention RTE meats, smoked salmon and fresh salmon, on cheese, on frozen vegetables, environmental surfaces	USDA/FDA GRAS approved. It is further accepted as a processing aid in Australia, New Zealand, Israel, Switzerland, The Netherlands (EU) Canada and others.
	PhageGuard S Salmonelex™	*Salmonella* spp. on fresh poultry	USDA and FDA GRAS. Processing aid approvals for USDA appear in 7120.1. It is further accepted as a processing aid in Canada, Australia, Israel and others.
	PhageGuard E	*Escherichia coli* O157 on beef carcasses, primals, subs and trimmings.	USDA and FDA approved
	Staphefect™ (Endolysin)	*Staphylococcus aureus* including MRSA on the human skin	Europe
Brimrose Technology Corporation www.brimrosetechno-logy.com	EnkoPhagum	*Salmonella* spp., *Shigella* spp. Enteropathogenic serotypes of *E. coli* *Staphylococcus* spp.	Former Soviet Union country of Georgia
	PYO Phage	*Staphylococcus* spp., *E. coli* *Streptococcus* spp. *Proteus* spp., *Pseudomonas* spp.	
	SES Phage	*Staphylococcus* spp. Enteropathogenic serotypes of *E. coli* *Streptococcus* spp.	
	Intesti Phage	*Shigella* spp., *Salmonella* spp., *Staphylococcu*s spp., *Proteus* spp., *E. coli* – different serotypes, *P. aeruginosa*	
	Fersisi Phage	*Staphylococcus* spp. *Streptococcus* spp.	
	Mono-phage	*Staphylococcus* spp. *E. coli*, S*treptococcu*s spp. *Enterococcus* spp. *P. aeruginosa*, *Proteus* spp.	
APS Biocontrol Ltd./UK www.apsbiocontrol.com	Biolyse™	Soft rot bacteria*: Erwinia, Pectobacterium, Pseudomonas* on potatoes	UK, Europe
Proteon Pharma-ceuticals SA/Poland www.proteonpharma.com	BAFASAL^®^	Eliminates human-pathogenic Salmonella in poultry farming	Ukraine
	BAFADOR^®^	Pseudomonas and Aeromonas infections in commercial aquaculture	

## 3. New trends in nutrition

We can observe a new trend in the use of bacteriophages to enhance the effect of probiotics order to fine-tune the microflora of both humans and animals and/or other microbiomes. For example, Intralytix Inc. has developed a patented platform technology PhageBiotix™ based on the use of lytic bacteriophages to specifically target the ‘problematic’ bacterial species before they can cause disease. Furthermore, this product is synergistic with traditional bacteria-based probiotics, and is therefore combined to form SuperBiotix™ line of products which contain both traditional bacteria-based probiotic cultures and PhageBiotix™ [63]. Another research regarding bacteriophage probiotics is carried out by a Polish company—Proteon Pharmaceuticals S.A. Their project aims at developing bacteriophage probiotic feed additives to limit the use of antibiotics in the poultry cultures [64].

## 4. Application of bacteriophages and phage-borne enzymes in the process of biosanitization of surfaces in the food industry

The formation of microbiological biofilms on the surfaces of equipment is one of the main problems in the food production plants. Bacterial biofilms are defined as aggregates of cells encased in a self-produced matrix of extracellular polymeric substances (EPS) and adherent to biotic or abiotic surfaces [65]. Bacterial cells that form biofilms are characterized by high resistance to adverse environmental conditions, antibiotics, and disinfectants [65]. In the fresh produce industry, the most of pathogenic bacteria included: *L. monocytogenes*, *Salmonella*, *E. coli*, *Yersinia* are able to adhere to plant tissues where they can grow forming biofilms [66]–[68]. The availability of sanitizers to these microorganisms is hindered due to intrinsic structure of vegetables. It making necessary to create preparations that are harmless to humans and eliminate biofilm on plant tissues. Bacteriophages give hope for creating a safe sanitizers for humans [66]. *Campylobacter jejuni* is one of the pathogenic bacteria that can form biofilms on the materials commonly used in industries (i.e. polyvinyl chloride and stainless steel). Lytic bacteriophages CP8 and CP30 isolated from the poultry excreta were used for preventing the formation of *C. jejuni* biofilm on glass Petri plates. The authors observed that phage treatment of each biofilm led to a reduction of 1.0–3.0 log CFU/cm^2^ in the viable count 24 hours after infection, compared with control nonbacteriophage-treated biofilm. The level of reduction and the possibility of developing resistance to phage varied depending on the biofilm-forming strain. *C. jejuni* strain PT14 was characterized by the ability to produce a far greater quantity of biofilm on glass than did *C. jejuni* strain NCTC 11168.In addition, among the bacteria that survived the bacteriophages treatment, no resistant *C. jejuni* cells remained in the PT14 biofilm treated by phages. In the case of *C. jejuni* 11168,among the cells that remained after phages treatment, 84% exhibited resistance to phage CP8 and 90% were resistant to phage CP30 [69].

*Listeria monocytogenes* is another pathogenic bacteria causing food poisoning and is able to form biofilm on the surfaces of conveyor belts, floor drains, stainless steel equipment, and product transportation racks. The efficiency of commercially available phage P100 was tested on the biofilm created by *L. monocytogenes*on the surface of stainless steel coupon. Twenty one strains representing 13 different serotypes were used in this research. Considerable differences were noticed in the ability of various strains of *L. monocytogenes* to form biofilms. A reduction of *L. monocytogenes* population at extracellular polymeric substances at a level of 5.4 log/cm^2^ was observed 24 hours after phage treatment of two-day-old biofilm formed on the surface of stainless steel coupon. Phage treatment of multilayer 1-week-old biofilm resulted in a reduction of biofilm cells by 3.5 log/cm^2^. The main conclusions that can be drawn from the above-described results are that the P100 phage is characterized by a wide range of *L. monocytogenes* hosts and shows considerable ability to reduce *L .monocytogenes* biofilm irrespective of the serotype or biofilm levels [70].

Biofilms are dynamic structures whose susceptibility to bacteriophages depends on the type of bacteriophage and its ability to produce enzymes that can degrade the biofilm structure and the availability of receptor sites for phage [71].

It seems that apart from bacteriophages the greatest potential for combating bacterial biofilms is exhibited by the enzymes produced by the bacteriophages (depolymerases and endolysins). Phage DP can degrade the cell-associated polysaccharides such as structural or capsular polysaccharides, as well as biofilm EPS, to facilitate cell adhesion of bacteriophages. Since the discovery and description of the phage DP for the first time in 1929, the activity of 160 putative bacteriophage DP from 143 bacteriophages has been described, including DP specific for *E.coli*, *E.amylovara*, *Azotobacter vinelandii*, *Vibrio cholerae* O139, and *Pseudomonas agglomerans*. Most of the studied DP were isolated from the *Myoviridae*, *Siphoviridae*, and *Podoviridae* family. Phage DP can occur as an integral part of the phage particles found mostly on tail fibers and base plates of bacteriophages. These enzymes may also appear in the form of soluble proteins secreted during the lysis of host cells. It was noticed that some bacteriophages are able to produce only one form of DP, while some (i.e. F1 and F29 phages specific to *E. coli*) are able to produce both forms of DP—connected and unconnected with phage particles [72]. DP activity is determined from the area of a constantly increasing halo zone surrounding the phage plaques. The halo effect is a result of excessive secretion of DP which diffuses into the medium and deprivesthe bacteria of the capsule during the stationary phase of their growth. Depending on their catalytic activity, phage DP may be classified into two groups, hydrolases (also known as polysaccharases) and lyases, which are further divided into different subclasses—for example, polysaccharases are divided into endoglucosidase, endogalactosidase, and endorhamnosidase, and lyases are divided into alginate and hyaluronan lyase [72],[73]. Most bacteriophages and phage-borne enzymes are characterized by high specificity [72]. In order to improve phage viability during application in the field, a carrier-phage system was developed that uses a non-pathogenic epiphytic bacterium, named ‘the carrier’. The ‘carrier’ protects the bacteriophages during processing and field applications. *Pantoea agglomerans* (*Enterobacter agglomerans*) have been described as a potential phage carrier against *E. amylovora*. This bacteria delivers and propagates the phages to the open blossoms, prior to the arrival of *E. amylovora*. The exopolysaccharide (EPS) layer, is the first cellular component which bacteriophages encounter during the infection of *P. agglomerans*, therefore, research on the interaction of *P. agglomerans* phages and biofilms should be performed [74]. The antimicrobial activity of DP produced by SF153b phage against biofilm formed by plant pathogen *Enterobacter agglomerans* was described. The bacterial biofilm was treated by the mixture of *E. agglomerans*-specific phage and phage polysaccharide DP for 180 minutes. At the end of treatment, a 1992-fold decline in the count of bacterial cells was observed in the biofilm. The biofilm exposure specifically to DP enzyme alone resulted in a 120-fold reduction of viable biofilm cells. A 61-fold decrease in the number of biofilm cells occurred when a mixture of phage and phage DP was used for treating the biofilm formed by resistant strain M53b. M53b EPS was sensitive to DP activity [75]. The synergistic effect of the application of bacteriophage DP and chlorine dioxide on the destruction of the structure of bacterial biofilm was observed. A *Klebsiella* sp. strain capable of forming biofilm was isolated from the instruments of the food processing plant. After 4 hours of exposing the biofilm to the bacteriophage DP, an 80% reduction of bacterial cells in the biofilm was achieved. The researchers also noticed that the treatment of *Klebsiella* biofilm with DP for 4 hours followed by a 30-minute treatment with chlorine dioxide resulted in the elimination of 92% of cells forming *Klebsiella* biofilm [76].

The second type of enzyme produced by bacteriophages that has potential applications in biosanitizationis endolysins. Endolysins are produced by bacteriophages during the terminal stage of their lytic cycle and allow the release of progeny virions through the degradation of peptidoglycan present in the cell wall [72].They can also be applied exogenously to destroy Gram-positive bacterial cells; Gram-negative bacteria possess an outer membrane that protects them from endolysins activity. Lysins are categorized into five different groups based on their cleavage sites in the peptidoglycan: glucosaminidases, lytic transglycosylases, muramidases, amidases, and endopeptidases [77]. Endolysin LysH5, produced by the *S. aureus* phage vB_SauS-phiIPLA88, was found to decrease staphylococcal counts by 1.0–3.0 log units in polystyrene-adhered biofilm, compared to the untreated control [78]. According to Oliviera et al (2014) for achieving good efficacy with endolysins against Gram-negative bacteria, an additional factor to disestablish the outer membrane by acting on the bacterial envelope is required [79]. Their study indicated that citric or malic acids are outer membrane permeabilizers and showed that the combination of lysin Lys68 with citric or malic acid resulted in a 3.0–5.0 log reduction in the cell concentration of *S.* Typhimurium LT2 biofilm after 2 hours of treatment [80]. The synergistic effect of LysK endolysin and DA7 depolymerase on staphylococcus biofilm has also been proven. Even very low concentrations of nano and micromolar mixtures of these two enzymes were effective in removing biofilm from polystyrene and glass surfaces [81]. The recently discovered endolysin LysCSA13 isolated from the virulent CSA13 phage also has great potential to combat *S. aureus* biofilms. LysCSA13 express strong antimicrobial activity against *S. aureus* strains at pH 7.0–9.0, 4.0–37.0 °C and in the presence of Ca^2+^ and Mn^2+^. Endolysin LysCSA13 is able to reduce biofilm by 80–90% on the surface of polystyrene, glass and stainless steel [82].

### 4.1. Multiple applications of bacteriophage endolysins in food preservation

The application of bacteriophage endolysins is envisaged at various stages of food production and processing: from agriculture to food packaging and detection of pathogens in food. There are two methods to apply endolysins extracellular polymeric substances to food. The first method involves the direct application of purified endolys into food or combining endolysin proteins with other agents used for food preservation [6]. For instance, a fragment of bacteriophage ΦH5 genome (gene *lys*H5) coding for *Staphylococcus*-specific endolys in was characterized. Purified endolysin was directly added to pasteurized milk previously contaminated by *S. aureus* at concentrations of 10^6^ and 10^3^ CFU/mL. The highest tested dose of endolysin (88 µg/mL) reduced the viable cells of bacteria to undetectable levels after 4 hours of the enzyme application. After 60 minutes of treatment, 88 µg/mL of endolysin resulted in a decrease of bacterial cells by 1 log unit below the control culture. The lower level of contamination required a proportionally lower dose of enzyme to complete the elimination of bacterial cells after 4 hours [80].The researchers also discovered that endolysin LysZ5 was specific for *Listeria* sp. and capable of destroying host cells at cold storage temperatures. They proved that LysZ5 reduced the population of *L. monocytogenes* by more than 4 log CFU/mL after a 3-hour incubation at 4 °C in soya milk [83].

Currently, there are many studies on food preservation based on high hydrostatic pressure (HHP). One of them showed that the combination of lysins and HHP technique was excellent in controlling food contamination by *L. monocytogenes*. The use of *L. monocytogenes*-specific endolysin PlyP825 at a concentration of 0.16 µg/mL led to a reduction of 0.2 log CFU, while the application of HHPs (300 MPa, 1 min, 30 °C) resulted in a reduction of 0.3 log CFU. The combinatory effect of HHP and endolysin reduced the number of *L. monocytogenes* by 5.5 log CFU. The synergistic effect of HHP and endolysin also enabled the effective application of lower pressure parameters (200 MPa, 2 min, 30 °C) [84].

The second promising method of applying bacteriophages to food involves the use of lysin-secreting recombinant bacteria [6]. Bacteriophage genes *ply118* and *ply511*encoding lysins specific to *L. monocytogenes* were cloned and expressed in *Lactococcus lactis.* The expression of the listerial lysin-encoding gene was found to be under the control of the lactoccocal promoter P32 [85].This allows adding recombinant starter cultures that can prevent the development of bacterial pathogens to the dairy products. The use of endolysins in food seems to be a safer solution than using only bacteriophages. To date, no endolysin-resistant strains have been identified [6].The development of resistance to endolysins is difficult for bacteria because these organisms would have to modify the structure of their cell wall.

## 5. Dual nature of bacteriophages in the dairy sector

Due to their ubiquitous presence and constant maintenance of bacterial populations, bacteriophages can contribute to huge financial losses in the industry. The presence of phages is especially undesirable in the industries where bacteria are used to produce a molecule or chemical compound. The undesirable presence of bacteriophages is most frequently recorded in the pharmaceutical, chemical, feed, probiotic, and food industries. In the dairy industry, bacteriophages destroy the fermentation process by the lysis of lactic acid bacteria (LAB). The most common starter cultures used in the dairy industry are *Lactococcus lactis*, *Streptococcus thermophilus*, *Leuconostoc* sp., and *Lactobacillus* sp. Bacteriophages capable of destroying starter cultures were identified to be belonging to the families *Caudovirales*, *Podoviridae*, and *Siphoviridae* [86].

There are many sources for phage contamination of fermented products, which include raw milk and whey powder [86],[87]. Raw milk is a natural LAB reservoir and thus also may possess LAB-specific bacteriophages. Starter LAB cultures can also be a source of bacteriophage infection. Temperate phages integrate their genetic material with the host's genetic material which can be passively transferred to bacterial progeny cells during the replication of bacterial cells. In the phage life cycle, a temperate bacteriophage of LAB may remain in the form of prophage for a longtime without leading to lysis of bacterial cells. Stress related to food processing can activate the prophage and trigger the lytic cycle leading to the death of the LAB cells during fermentation [86]. 30 commercial strains and dairy-isolated *Lactobacillus casei*, *Lactobacillus paracasei*, or *Lactobacillus rhamnosus* strains were tested for the presence of prophages in the genome. Out of the 30 tested strains, 25 possessed inducible prophages in the genome [88]. Therefore, the majority of commercially available starter cultures are screened for the presence of phages before they reach customers. The other important sources of bacteriophage contamination are air and surface in dairy plants. This is a major problem in cheese factories because whey separation often leads to aerosol-borne phages and thus contamination of the factory environment. Therefore, whey proteins used for recycling into cheese matrices are exposed to UV radiation, thermal treatment, and membrane filtration. These methods are aimed to eliminate the risk of fermentation failure during recycling of whey [87]. Bacteriophages can also be helpful in the dairy industry. Due to their high specificity, phages can be used to destroy pathogens, for example, *Staphylococcus* bacteria often found in dairy products. The *Staphylococcus*-contaminated pasteurized milk was exposed to three lytic phages: SA, SANF, and SA2. All three phages exhibited an increased ability to reduce *Staphylococcus* from 4 to 6 hours after infection [89]. Due to the ubiquitous nature of bacteriophages that infect LAB starter cultures in a production environment, it is difficult to completely eliminate potential infection and prevent the failure of fermentation. However, the risks of fermentation failure can be reduced by the following:

- Use of effective disinfectants—peracetic acid and sodium hypochlorite are one of the most efficient biocides tested on LAB-specific phages. Very little data are available on the efficiency of ozone treatment and UV light irradiation on phages in the industrial environment.
- A simple and old method that is still used in production plants (mainly cheese plant)is starter/strain rotation.
- Genetic engineering methods that allow the construction of bacteriophage-resistant strains of LAB are promising.

### 5.1. Method of phage detection in dairy products

The detection of bacteriophages in the individual ingredients is important for maintaining the fermentation process. For phage detection, the classical microbiological methods, such as plaque test or acidification monitoring, are used. These methods are quantitative and sensitive, but time-consuming. It is also possible to use molecular biological methods, including qPCR and classic PCR, to detect bacteriophages in raw milk. In classical PCR, a complete analysis takes several hours, and the lowest level of phage contamination that can be detected by this method is 10^3^ PFU/mL. In contrast to classical PCR, the qPCR method using fluorescent techniques allows monitoring the amount of the reaction product during its lifetime. As a result, the bacteriophage detection can be done quickly. Molecular methods of phage detection may be too specific which means that not all types of phages found in a given product can be detected. An additional method that can be successfully used for phage detection in the dairy industry is flow cytometry. This method has been effectively used for skimmed milk-enriched culture infected by bacteriophages. A condition for obtaining a real result is to remove the fat particles that could block the cytometer before the test. In this method, mass changes and the interruption of cell division are monitored through the massive death of bacterial cells [90].

## 6. Conclusions

Bacteriophages can be widely applied in the food industry. They can be used for the protection of food products at the pre- and postharvest stage, as preservatives to extend the expiry date of food products, and to keep clean the surfaces of equipment used in production plants. In the dairy industry, the dual nature of bacteriophages is considered a huge challenge. Their negative impact on starter cultures causes huge financial losses, while their high specificity allows using them to destroy pathogenic bacteria found in dairy products, without any negative impact on starter cultures. Due to the many limitations associated with the use of bacteriophages in the industry, including resistance and low tolerance to unfavorable environmental conditions, attempts are made to use specific enzymes produced by bacteriophages for food protection. In addition, bacteriophages are used as one of the components of biosensors for the rapid detection of pathogens in food. The use of bacteriophages in foods seems to be as effective as antibiotics, but safer and more ecological.

## References

[1] Hendrix WR (2002). Bacteriophages: evolution of the majority. Theor Popul Biol.

[2] Hietala V, Horsma-Heikkinen J, Carron A (2019). The removal of endo- and enterotoxins from bacteriophage preparations. Front Microbiol.

[3] Sarhan WA, Azzazy HM (2015). Phage approved in food, why not as a therapeutic?. Expert Rev Anti Infect Ther.

[4] Górski A, Międzybrodzki R, Borysowski J (2012). Phage as a modulator of immune responses: practical implications for phage therapy. Adv Virus Res.

[5] Wittebole X, Roock De S, Opa M (2014). Historical overview of bacteriophage therapy as an alternative to antibiotics for the treatment of bacterial pathogens. Virulence.

[6] Kazi M, Annapure US (2016). Bacteriophage biocontrol of foodborne pathogens. J Food Sci Technol.

[7] Gilmore BF (2012). Bacteriophages as anti-infective agents: recent developments and regulatory challenges. Expert Rev Anti Infe Ther.

[8] Fernández L, Gutiérrez D, Rodríguez A (2018). Application of bacteriophages in the agro-food sector: a long way toward approval. Front Cell Infect Microbiol.

[9] Balogh B, Jones JB, Iriarte FB (2010). Phage therapy for plant disease control. Curr Pharm Biotechno.

[10] Civerolo EL, Kiel HL (1969). Inhibition of bacterial spot of peach foliage by *Xanthomonas pruni* bacteriophage. Phytopathology.

[11] Eman OH, El-Meneisy Afaf ZA (2014). Biocontrol of halo blight of bean caused by pseudomonas phaseolicola. Int J Virol.

[12] Fujiwara A, Fujisawa M, Hamasaki R (2011). Biocontrol of ralstonia solanacearum by treatment with lytic bacteriophages. Appl Environ Microbiol.

[13] Born Y, Bosshard L, Duffy B (2015). Protection of Erwinia amylovora bacteriophage Y2 from UV-induced damage by natural compounds. Bacteriophage.

[14] Zaccardelli M, Saccardi A, Gambin E (1992). *Xanthomonas campestris* pv. *pruni* bacteriophages on peach trees and their potential use for biological control. Plant Pathogenic Bacteria 8^th^ International Conference.

[15] Balogh B, Canteros BI, Stall RE (2008). Control of citrus canker and citrus bacterial spot with bacteriophages. Plant Dis.

[16] Balogh B, Jones JB, Iriarte FB (2010). Phage therapy for plant disease control. Curr Pharm Biotechno.

[17] Leverentz B, Conway WS, Alavidze Z (2001). Examination of bacteriophage as a biocontrol method for *Salmonella* on fresh-cut fruit: a model study. J Food Protect.

[18] Szczepankowska A (2012). Role of CRISPR/cas system in the development of bacteriophage resistance. Adv Virus Res.

[19] Koskella B, Brockhurs MA (2014). Bacteria–phage coevolution as a driver of ecological and evolutionary processes in microbial communities. FEMS Microbiol Rev.

[20] Carrillo LC, Atterbury JR, El-Shibiny A (2005). Bacteriophage therapy to reduce *Campylobacter jejuni* colonization of broiler chickens. Appl Environ Microb.

[21] Wagenaar AJ, Van Bergen M, Mueller M (2005). Phage therapy reduces *Campylobacter jejuni* colonization in broilers. Vet Microbiol.

[22] Arthur MT, Kalchayanand N, Agga EG (2017). Evaluation of bacteriophage application to cattle in lairage at beef processing plants to reduce *Escherichia coli* O157:H7. Prevalence on hides and carcasses. Foodborne Pathog Dis.

[23] Wall KS, Zhang J, Rostagno HM (2010). Phage therapy to reduce preprocessing *Salmonella* infections in market-weight swine. Appl Environ Microb.

[24] Bach JS, Johnson PR, Stanford K (2009). Bacteriophages reduce *Escherichia coli* O157:H7 levels in experimentally inoculated sheep. Can J Animal Sci.

[25] Huanga K, Nitin N (2019). Edible bacteriophage based antimicrobial coating on fish feed for enhanced treatment of bacterial infections in aquaculture industry. Aquaculture.

[26] Rivas L, Coffey B, McAuliffe O (2010). In vivo and ex vivo evaluations of bacteriophages e11/2 and e4/1c for use in the control of Escherichia coli O157:H7. App Environ Microb.

[27] Hussain MA, Liu H, Wang Q (2017). Use of encapsulated bacteriophages to enhance farm to fork food safety. Crit Rev Food Sci.

[28] Murthy K, Engelhardt R (2012). Encapsulated bacteriophage formulation. United States Patent 2012/0258175 A1.

[29] Stanford K, Mcallister AT, Niu DY (2010). Oral delivery systems for encapsulated bacteriophages targeted at Escherichia coli O157:H7 in Feedlot Cattle. J Food Protect.

[30] Saez AC, Zhang J, Rostagno MH (2011). Direct feeding of microencapsulated bacteriophages to reduce *Salmonella* colonization in pigs. Foodborne Pathog Dis.

[31] Ma Y, Pacan CJ, Wang Q (2008). Microencapsulation of bacteriophage felix O1 into chitosan-alginate microspheres for oral delivery. Appl Environ Microb.

[32] EFSA (European Food Safety Authority), ECDC (European Centre for Disease Prevention and Control) (2017). The European Union summary report on trends and sources of zoonoses, zoonotic agents and food-borne outbreaks in 2016. EFSA J.

[33] Word Health Organzation (2019). Food safety. https://www.who.int/news-room/fact-sheets/detail/food-safety.

[34] Moye ZD, Woolstone J, Sulakvelidze A (2018). Bacteriophage Applications for Food Production and Processing. Viruses.

[35] Endersen L, O'Mahony J, Hill C (2014). Phage Therapy in the Food Industry. Annu. Rev Food Sci Technol.

[36] de Melo AG, Levesque S, Moineau S (2018). Phages as friends and enemies in food processing. Curr Opin Biotechnol.

[37] Atterbury RJ, Connerton PL, Dodd CE (2003). Application of host-specific bacteriophages to the surface of chicken skin leads to a reduction in recovery of *Campylobacter jejuni*. Appl Environ Microb.

[38] Goode D, Allen VM, Barrow PA (2003). Reduction of experimental *Salmonella* and *Campylobacter* contamination of chicken skin by application of lytic bacteriophages. Appl Environ Microb.

[39] Bigwood T, Hudson JA, Billington C (2009). Influence of host and bacteriophage concentrations on the inactivation of food-borne pathogenic bacteria by two phages. FEMS Microbiol Lett.

[40] Orquera S, Golz G, Hertwig S (2012). Control of *Campylobacter* spp. and *Yersinia enterocolitica* by virulent bacteriophages. J Mol Genet Med.

[41] O'Flynn G, Ross RP, Fitzgerald GF (2004). Evaluation of a cocktail of three bacteriophages for biocontrol of *Escherichia coli* O157:H7. Appl Environ Microb.

[42] Abuladze T, Li M, Menetrez MY (2008). Bacteriophages reduce experimental contamination of hard surfaces, tomato, spinach, broccoli, and ground beef by *Escherichia coli* O157:H7. Appl Environ Microb.

[43] Sharma M, Patel JR, Conway WS (2009). Effectiveness of bacteriophages in reducing *Escherichia coli* O157:H7 on fresh-cut cantaloupe and lettuce. J Food Prot.

[44] Carter CD, Parks A, Abuladze T (2012). Bacteriophage cocktail significantly reduced *Escherichia coli* O157H:7contamination of lettuce and beef, but does not protect against recontamination. Bacteriophage.

[45] Boyacioglu O, Sharma M, Sulakvelidze A (2013). Biocontrol of *Escherichia coli* O157: H7 on fresh-cut leafy greens. Bacteriophage.

[46] Viazis S, Akhtar M, Feirtag J (2011). Reduction of *Escherichia coli* O157:H7 viability on leafy green vegetables by treatment with a bacteriophage mixture and trans-cinnamaldehyde. Food Microbiol.

[47] Patel J, Sharma M, Millner P (2011). Inactivation of *Escherichia coli* O157:H7 attached to spinach harvester blade using bacteriophage. Foodborne Pathog Dis.

[48] Carlton RM, Noordman WH, Biswas B (2005). Bacteriophage P100 for control of *Listeria monocytogenes* in foods: genome sequence, bioinformatic analyses, oral toxicity study, and application. Regul Toxicol Pharm.

[49] Holck A, Berg J (2009). Inhibition of *Listeria monocytogenes* in cooked ham by virulent bacteriophages and protective cultures. Appl Environ Microbiol.

[50] Soni KA, Nannapaneni R, Hagens S (2010). Reduction of *Listeria monocytogenes* on the surface of fresh channel catfish fillets by bacteriophage listex p100. Foodborne Pathog Dis.

[51] Soni KA, Desai M, Oladunjoye A (2012). Reduction of *Listeria monocytogenes* in queso fresco cheese by a combination of listericidal and listeriostatic GRAS antimicrobials. Int J Food Microbiol.

[52] Chibeu A, Agius L, Gao A (2013). Efficacy of bacteriophage LISTEX™ P100 combined with chemical antimicrobials in reducing *Listeria monocytogenes* in cooked turkey and roast beef. Int J Food Microbiol.

[53] Figueiredo ACL, Almeida RCC (2017). Antibacterial efficacy of nisin, bacteriophage P100 and sodium lactate against *Listeria monocytogenes* in ready-to-eat sliced pork ham. Braz J Microbiol.

[54] Guenther S, Loessner MJ (2011). Bacteriophage biocontrol of *Listeria monocytogenes* on soft ripened white mold and red-smear cheeses. Bacteriophage.

[55] Bigot B, Lee WJ, McIntyre L (2011). Control of *Listeria monocytogenes* growth in a ready-to-eat poultry product using a bacteriophage. Food Microbiol.

[56] Modi R, Hirvi Y, Hill A (2001). Effect of phage on survival of *Salmonella* Enteritidis during manufacture and storage of cheddar cheese made from raw and pasteurized milk. J Food Protect.

[57] Leverentz B, Conway WS, Camp MJ (2003). Biocontrol of *Listeria monocytogenes* on fresh-cut produce by treatment with lytic bacteriophages and a bacteriocin. Appl Environ Microbiol.

[58] Whichard JM, Sriranganathan N, Pierson FW (2003). Suppression of *Salmonella* growth by wild-type and large-plaque variants of bacteriophage Felix O1 in liquid culture and on chicken frankfurters. J Food Prot.

[59] Guenther S, Herzig O, Fieseler L (2012). Biocontrol of *Salmonella* Typhimurium in RTE foods with the virulent bacteriophage FO1-E2. Int J Food Microbiol.

[60] Spricigo DA, Bardina C, Cortés P (2013). Use of a bacteriophage cocktail to control *Salmonella* in food and the food industry. Int J Food Microbiol.

[61] Farber JM, Peterkin PI (1991). *Listeria monocytogenes*, a foodborne pathogen. Microbiol Rev.

[62] Leistner L, Gorris LGM (1995). Food preservation by hurdle technology. Trends Food Sci Technol.

[63] Phages as probiotics. http://intralytix.com/index.php?page=pro.

[64] Proteon Pharmaceuticals. https://www.proteonpharma.com.

[65] Schmelcher M, Loessner JM (2016). Bacteriophage endolysins: applications for food safety. Curr Opin Biotechnol.

[66] Gutiérrez D, Rodríguez-Rubio L, Martíne B (2016). Bacteriophages as weapons against bacterial biofilms in the food industry. Front Microbiol.

[67] Da Silva Felício MT, Hald T, Liebana E (2015). Risk ranking of pathogens in ready-to-eat unprocessed foods of non-animal origin (FoNAO) in the EU: initial evaluation using outbreak data (2007–2011). Int J Food Microbiol.

[68] Beuchat LR (2002). Ecological factors influencing survival and growth of human pathogens on raw fruits and vegetables. Microbes Infect.

[69] Siringan P, Connerton PL, Payne RJ (2011). Bacteriophage-mediated dispersal of *Campylobacter jejuni* biofilms. Appl Environ Microb.

[70] Soni KA, Nannapaneni R, Hagens S (2010). Reduction of *Listeria monocytogenes* on the surface of fresh channel catfish fillets by bacteriophage listex p100. Foodborne Pathog Dis.

[71] Sutherland IW, Hughes KA, Skillman LC (2004). The interaction of phage and biofilms. FEMS Microbiol Lett.

[72] Maszewska A (2015). Phage associated polysaccharide depolymerases–characteristics and application. Postep Hig Med Dos.

[73] Drulis-Kawa Z, Majkowska-Skrobek G, Maciejewska B (2015). Bacteriophages and phage-derived proteins—application approaches. Curr Med Chem.

[74] Lehman SM (2007). Development of a bacteriophage-based biopesticide for fire blight. PhD Thesis.

[75] Hughes KA, Sutherland IW, Jones MV (1998). Biofilm susceptibility to bacteriophage attack: the role of phage-borne polysaccharide depolymerase. Microbiology.

[76] Chai Z, Wang J, Tao S (2014). Application of bacteriophage-borne enzyme combined with chlorine dioxide on controlling bacterial biofilm. LWT Food Sci Technol.

[77] Love JM, Bhandari D, Dobson CR (2018). Potential for bacteriophage endolysins to supplement or replace antibiotics in food production and clinical care. Antibiotics.

[78] Gutierrez D, Ruas-Madiedo P, Martınez B (2014). Effective removal of Staphylococcal biofilms by the endolysin LysH5. PloS One.

[79] Oliveira H, Thiagarajan V, Walmagh M (2014). A thermostable *Salmonella* phage endolysin Lys68, with broad bactericidal properties against gram-negative pathogens in presence of weak acids. PloS One.

[80] Obeso MJ, Martínez B, Rodríguez A (2008). Lytic activity of the recombinant staphylococcal bacteriophage ΦH5 endolysin active against *Staphylococcus aureus* in milk. Int J Food Microbiol.

[81] Olsen NMC, Thiran E, Hasler T (2018). Synergistic removal of static and dynamic *Staphylococcus aureus* biofilms by combined treatment with a bacteriophage endolysin and a polysaccharide depolymerase. Viruses.

[82] Yoyeon Ch, Son B, Ryu S (2019). Effective removal of staphylococcal biofilms on various food contact surfaces by Staphylococcus aureus phage endolysin LysCSA13. Food Microbiol.

[83] Zhang H, Bao H, Billington C (2012). Isolation and lytic activity of the *Listeria* bacteriophage endolysin LysZ5 against *Listeria monocytogenes* in soya milk. Food Microbiol.

[84] Van Nassau TJ, Lenz CA, Scherzinger AS (2017). Combination of endolysins and high pressure to inactivate *Listeria monocytogenes*. Food Microbiol.

[85] Gaeng S, Scherer S, Neve H (2000). Gene cloning and expression and secretion of *Listeria monocytogenes* bacteriophage-lytic enzymes *in Lactococcus lactis*. Appl Environ Microb.

[86] Garneau EJ, Moineau S (2001). Bacteriophages of lactic acid bacteria and their impact on milk fermentations. Microb Cell Fact.

[87] Atamer Z, Samtlebe M, Neve H (2013). Review: elimination of bacteriophages in whey and whey products. Front Microbiol.

[88] Mercanti D, Carminati D, Reinheimer JA (2011). Widely distributed lysogeny in probiotic lactobacilli represents a potentially high risk for the fermentative dairy industry. Int J Food Microbiol.

[89] Tahir A, Asif M, Abbas Z (2017). Three bacteriophages SA, SA2 and SNAF can control growth of milk isolated Staphylococcal species. Pak J Zool.

[90] Singh A, Poshtiban S, Evoy S (2013). Recent advances in bacteriophage based biosensors for food-borne pathogen detection. Sensors.

